# Melatonin’s Antineoplastic Potential Against Glioblastoma

**DOI:** 10.3390/cells9030599

**Published:** 2020-03-03

**Authors:** Enrico Moretti, Gaia Favero, Luigi Fabrizio Rodella, Rita Rezzani

**Affiliations:** 1 Anatomy and Physiopathology Division, Department of Clinical and Experimental Sciences, University of Brescia, Viale Europa 11, 25123 Brescia, Italy; segreteria.anatomia@unibs.it (E.M.); gaia.favero@unibs.it (G.F.); luigi.rodella@unibs.it (L.F.R.); 2 Interdipartimental University Center of Research “Adaption and Regeneration of Tissues and Organs-(ARTO)”, University of Brescia, 25123 Brescia, Italy

**Keywords:** glioblastoma, melatonin, antineoplastic effect

## Abstract

Glioblastoma (GBM) is one of the most intransigent and aggressive brain tumors, and its treatment is extremely challenging and ineffective. To improve patients’ expectancy and quality of life, new therapeutic approaches were investigated. Melatonin is an endogenous indoleamine with an incredible variety of properties. Due to evidence demonstrating melatonin’s activity against several cancer hallmarks, there is growing interest in its use for preventing and treating cancer. In this review, we report on the potential effects of melatonin, alone or in combination with anticancer drugs, against GBM. We also summarize melatonin targets and/or the intracellular pathways involved. Moreover, we describe melatonin’s epigenetic activity responsible for its antineoplastic effects. To date, there are too few clinical studies (involving a small number of patients) investigating the antineoplastic effects of melatonin against GBM. Nevertheless, these studies described improvement of GBM patients’ quality of life and did not show significant adverse effects. In this review, we also report on studies regarding melatonin-like molecules with the tumor-suppressive properties of melatonin together with implemented pharmacokinetics. Melatonin effects and mechanisms of action against GBM require more research attention due to the unquestionably high potential of this multitasking indoleamine in clinical practice.

## 1. Introduction

### 1.1. Glioblastoma

Glioblastoma (GBM) is the most aggressive and common primary brain tumor in adults, with an incidence rate of 5–8 per 100,000 habitants, and it represents nearly 54% of all diagnosed gliomas. GBM has poor prognosis with an average life expectancy of less than one year after diagnosis, due to the high rate of tumor recurrences [1,2]. Males show a GBM incidence of 1.6 times higher as compared to females, and they have a significantly higher frequency of primary GBM. However, secondary GBM frequency is higher in females [2,3]. GBM contains a subpopulation of glioma stem-like cells (GSCs) that grow as spheres and induce an increase in the tumor-propagating and self-renewing capacity [4,5,6]. Numerous studies reported that these GSCs play a particularly essential role in maintaining tumor growth and recurrence [7,8,9]. According to these observations, therapeutic approaches that do not eradicate GSCs are more likely to fail. To date, the standard therapy is radiotherapy and adjuvant chemotherapy with temozolomide (TMZ) [10]. TMZ is an oral alkylating agent capable of entering the cerebrospinal fluid and central nervous system, which contributes to improving the overall survival rate of patients with GBM [10,11]. Other drugs were tried, for example, bevacizumab and cilengitide. The effects of bevacizumab, an anti-angiogenetic monoclonal antibody, versus GBM were studied with the hopes of improving the efficacy of chemotherapy. To date, results show an increase in the progression-free survival without an increase in the overall survival [12]. Cilengitide, an integrin inhibitor, is another molecule studied as a therapy against GBM, yet there is no evidence concerning an increase in the overall survival or progression-free survival [13].

Unfortunately, compelling evidence indicates that the treatment of patients with GBM is extremely challenging, as complete surgical resection of the tumor is very difficult, and most GBMs tend to recur after chemotherapy and radiation therapy regimens [14,15]. Hence, some researches focused on novel co-therapeutic strategies, preferably based upon natural resources, to provide anticancer compounds eligible for clinical application. Various studies described the effects of melatonin against GBM, which is not only a recognized antioxidant molecule, but evidence is revealing its anticancer effects. Melatonin is a promising candidate to overcome multidrug resistance in the treatment of GBM [16,17,18]. Additionally, the dipeptide carnosine (β-alanyl-l-histidine) demonstrated antineoplastic effects not only limited to proliferation and cell-cycle control, but also having the ability to decrease the migration of GBM cells [19,20]. Another possible approach to anticancer therapy is to artificially produce new tumor-specific molecules. The formulation of peptides specific for membrane receptors overexpressed or uniquely expressed in GBM cells seems to be a promising therapeutic approach; however, to date, results show poor and unsatisfactory outcomes [21].

These routes provide valuable and distinctive strategies for improving GBM treatment, one of the most untreatable and deadly malignant diseases. Further studies are required for obtaining a coherent picture and detailed knowledge of tumor membrane receptors and the underlying intracellular signaling pathways involved in GBM formation and progression. The standard of care should evolve to include patients’ maximum safety during resection with concurrent and adjuvant chemotherapy. Future studies are required that focus on the develop of new therapeutic molecules and strategies, various types of combination therapies, and dose-finding trials.

### 1.2. Melatonin

Melatonin, an endogenous indoleamine with numerous properties and functions, is synthesized from l-tryptophan by the pineal gland and potentially in any tissue [22,23]. Melatonin possesses lipophilic and hydrophilic properties, which permit its receptor-independent transfer into almost all tissues and fluids. Melatonin, through both receptor-dependent and receptor-independent signaling pathways, activates a broad spectrum of molecular pathways. For example, melatonin acts as a natural chronobiotic substance with immune-enhancing properties, as well as having the ability to reduce oxidative stress [24,25,26]. It is well established that melatonin possesses anti-inflammatory and antioxidant activities, as well as having an influence on metabolism, the sleep–wake cycle, and reproduction [27,28,29,30]. The antioxidant activity of melatonin is observed through the prevention of free radical formation, as well as the capacity to upregulate antioxidant enzyme activity and downregulate pro-oxidant enzymes [31,32,33]. Furthermore, in cancer cells, melatonin presents a paradoxical pro-oxidant effect, leading to cell death [34,35,36]. Therefore, melatonin may inhibit cancer development by increasing the induction of oxidative stress in tumor cells [37]. As concluded by several studies, melatonin seems to recognize a cell’s context (normal or tumoral) and modulates the appropriate actions, thus behaving akin to a “smart killer” [34,38]. This aspect of melatonin is fundamental in oncological therapies; however, an understanding of how such a paradoxical behavior occurs is needed from future studies.

Melatonin has three different receptors that belong to the family of G protein-coupled transmembrane receptors: human melatonin receptor 1 (MT1), 2 (MT2), and 3 (MT3) [39,40,41]. A study by Dillon et al. [42] indicated that breast cancer cells have higher MT1 expression than normal cells, thus supporting a different susceptibility of cancer cells to melatonin. Despite the existence of melatonin receptors, many of the melatonin actions are mainly mediated through receptor-independent activities, such as antioxidant, anti-inflammatory, and mitochondrial activity [43,44,45,46]. There may also be melatonin binding sites in the nucleus of some cells that serve to synergistically influence the activity of antioxidant enzymes [47].

In recent years, an increasing number of studies investigated the abilities of melatonin to modulate several cancer cell processes, including proliferation, differentiation, invasion, and apoptosis [48,49,50]. Melatonin’s anticancer activity was described in several cancer types, such as ovarian, lung, breast, colon, and brain cancers [51,52,53,54,55]. Notably, melatonin may be able to reduce the proliferation of GBM-inducing cells (GICs), stem-like cells isolated from GBM specimens, as well as their self-renewal and clonogenic capability, modulating different biological processes and signaling [16,18]. In addition, no significant side effects were described with the use of melatonin in animal and human studies, even in extremely high doses [56,57]. Andersen et al. [57] reported only mild adverse effects, such as dizziness, sleepiness, nausea, and headaches.

In this review, we analyze the current evidence of melatonin effects against GBM and GSCs, both in vitro and in vivo, as a promising multitasking indoleamine for improving the efficacy of GBM therapy. It is worth mentioning that the benefits of melatonin for cancer recovery in clinical trials are debatable, noting that this multitasking indoleamine is mainly used as an adjuvant molecule to reduce chemotherapy or radiotherapy side effects and to improve patients’ sleep [58,59]. Nevertheless, these advantageous effects of melatonin as an adjuvant therapeutic are important and should not be underestimated during chemotherapy-related illness because such effects may substantially improve patient’s quality of life.

## 2. In Vitro Evidence of Melatonin Effects against Glioblastoma

As previously reported, GSCs are a cell subpopulation responsible for GBM tumor development, progression, relapse, and therapeutic resistance. Therefore, the eradication of GSCs and GICs is essential to achieve a long-lasting tumor remission. Martín et al. [60] evaluated the effects of melatonin alone in cells obtained from acute cell dissociation of human GBM postsurgical specimens. The authors found that melatonin treatment significantly reduced tumor cell proliferation and induced a decrease in self-renewal and clonogenic ability accompanied by a reduction in the expression of stem-cell markers, such as the transmembrane glycoprotein cluster of differentiation 133 (CD133). Interestingly, in the same study, similar effects did not appear during melatonin treatment of normal human neural cells, confirming, as previously reported, that cytotoxic effects of this indoleamine are specific for cancer cells [60]. Moreover, GBM cells treated with melatonin showed cell death with ultrastructural features of autophagy and a progressive accumulation of autophagosome vacuoles that led to the cellular membrane and organelle disruption.

Mitochondria are the main source of reactive oxygen species (ROS) production and are a known target of melatonin [61,62]. As previously reported, melatonin presented a pro-oxidant effect in cancer cells [34,35,36]. A high concentration of ROS can damage mitochondrial and nuclear DNA and modify the expression of oncogenes and tumor suppressor genes [63,64]. In the hopes of blocking tumor growth, Franco et al. [35] studied the effects of 3 mM melatonin on human GBM cells and observed a reduced expression of mitochondrial transcription factor A (TFAM), which is an essential protein that maintains mitochondrial DNA (mtDNA) integrity [65,66]. Moreover, melatonin selectively induced a surprisingly concentration-dependent increase in ROS production and delayed cell-cycle progression in GBM cancer cells as compared to the control group [35], while the presence of high levels of mitochondria-synthesized melatonin appears to be toxic for GBM and GSCs [67]. This effect of melatonin on ROS production may be due to the altered expression of respiratory chain genes, which could depend on the activity of the transcription factors TFAM, TFB1M, and TFB2M [35].

The concentration of melatonin required to inhibit cell growth in several tumor types is higher compared to its endogenous concentration in the bloodstream. In fact, pharmacological concentrations of this indoleamine are necessary to reduce neoplastic activity [68]. As described by Hevia et al. [69], the intracellular concentration of melatonin depend on the cell type; if the intracellular concentration of melatonin is high, its anticancer properties are improved. Together with the pharmacological concentration, the incubation time is crucial for melatonin therapeutic effects. It was observed that an incubation time less than 72 h did not induce effects in cell viability or intracellular ROS content [35]. Very few studies analyzed in detail if melatonin effects were receptor-mediated or -independent. Thus, more studies focused on these aspects could help to clarify melatonin’s mechanism of action against GBM. Interestingly, a study evaluating melatonin’s antineoplastic mechanism of action, carried out by Martín et al. [70], used both pertussis toxin (200 ng/mL), an inhibitor of G-coupled receptors, and luzindole (10 Amol/L), an MT1 and MT2 antagonist, and they observed that melatonin was effective in reducing glioma cell proliferation, confirming that melatonin may act through different molecular pathways. Specifically, the melatonin membrane receptor dissociation constant (K_d_) is in the nanomolar range. As a result, such low concentrations of melatonin do not appear to have any significant effect on rat glioma cells. Therefore, Martín et al. [70] evaluated the possible mediation of melatonin membrane receptors in its antiproliferative effect using high doses (1 mM) of melatonin, observing that melatonin binding to its membrane receptors did not mediate inhibition of GBM cell proliferation. The direct and indirect antioxidant activities of melatonin also play a significant role in contrasting GBM cells. Rat glioma cells show basal activation of nuclear factor κB (NF-κB) and activator protein-1 (AP-1), both transcription factors regulated by cellular oxidative stress. The treatment with 1 mmol/L melatonin reduced NF-κB activation, while no effects on the basal activation of AP-1 were described [70].

In the tumor microenvironment, infiltrating immune cells were shown to interact with tumor progression [71], and GBM shows an abundant population of infiltrating monocytes/macrophages that constitute up to 40% of a tumor’s mass and provide protection from immune surveillance [72]. The reduction of monocyte-related tumor-promotion effects in GBM could be a potentially useful antineoplastic strategy. With this in mind, Lai et al. [73] reported that in vitro melatonin supplementation reduced monocyte adhesion molecules, as well as monocyte chemoattractant chemokine CCL2 expression in GBM, consequently attenuating monocyte recruitment and activity. Importantly, Lai et al. [73] also observed that administration of melatonin increased the expression of sirtuin 1, which, in turn, reduced the expression of monocyte adhesion molecules.

Notably, one of the key features of melatonin in anticancer therapy is its epigenetic activity [74,75]. Melatonin can alter the fate of a neoplastic cell by downregulating oncogenes and upregulating oncosuppressive genes and interacting with intracellular signaling pathways, for example, selectively inducing cancer cell apoptosis [76,77,78]. As previously described in other cancerous cells, mitochondrial membrane depolarization increased in cells incubated with melatonin, thus indicating the release of cytochrome c and other pro-apoptotic factors, activating the cell death pathway [61,79,80,81]. Melatonin can influence transcription factors, such as the c-Jun N-terminal kinase (JNK) pathway, and it can activate caspase-3, inducing cell death [82]. Melatonin treatment is also involved in modifying the expression of another important transcription factor, nuclear factor erythroid 2-related factor (Nrf2), which interacts with the expression of several antioxidant and anti-inflammatory genes [76,83,84].

The downregulation of enhancer of zeste 2 polycomb repressive complex 2 subunit (*EZH2*) may in part explain the inhibitory effect of melatonin on GSCs. Zheng et al. [85], studying the knockdown of *EZH2* in GBM cells, observed a significant downregulation of Notch1, a transmembrane protein with an important role in cell embryonic and postnatal development [85,86,87,88] that is overexpressed in many cancer types, like breast, lung, pancreatic, and colon cancer [88]. The depletion of *EZH2* also reduced the quantity and self-renewal activity of GSCs. The treatment of GBM cells with melatonin induced remarkable growth inhibition in GBM, showing the role of melatonin in the inhibition of the *EZH2*–Notch1 signaling axis and its potential therapeutic significance [85].

It is known that the proprieties of cancerous cells are affected by surrounding environmental conditions. In particular, hypoxia is a well-characterized component of GBM microenvironment and a crucial factor in tumor aggressiveness and resistance to therapy [89,90]. In the study by Zhang et al. [91], hypoxic GBM cells showed an invasiveness increased more than two-fold compared with the respective controls under normoxia. Melatonin was observed to have anti-migratory and anti-invasive action in GBM not only under normoxia, but also under hypoxia [92]. Melatonin influenced hypoxia-inducible factor-1α (HIF-1α), a major oncogenic factor playing an essential role in tumor invasion and angiogenesis under hypoxia [93], as well as the activity of the enzymes responsible for the degradation of HIF-1α [91].

### Melatonin and Chemotherapy

One of the main problems in GBM pharmacologic therapy is chemoresistance. The main goals for improving patients’ survival and quality of life concerning co-therapy are to improve the cytotoxic effects of chemotherapy, while reducing chemoresistance and chemotherapeutic side effects. Clinical and experimental studies showed that melatonin enhanced the antineoplastic activity of chemotherapy in various cancer types. This, along with significantly reducing chemotherapy side effects, improves the patient’s quality of life [36,53,59]. McConnell et al. [94] studied the effects of the association of melatonin to TMZ in cultured GBM cells and GSCs. They also studied TMZ association with other antioxidants, alpha-lipoic acid and vitamin D3, which are widely available via an oral route, and which can readily cross the blood–brain barrier. Notably, each molecule decreased the proliferation of GBM cells. Vitamin D3 showed the least dramatic effect [95], and higher doses could be necessary for more significant results; additionally, blood levels of 375 nmol/L vitamin D3 resulted in hypervitaminosis [96]. Interestingly, melatonin showed a robust anti-proliferative activity both alone and combined with TMZ. Surprisingly, alpha-lipoic acid showed the most pertinent results with significant anti-proliferative activity both alone and in combination with TMZ. Nevertheless, even if several studies described a delay in cancer cell proliferation and apoptosis under melatonin-treatment, there is no evidence of a clear cancer regression with a net reduction in the GBM neoplastic mass using melatonin alone in clinical trials. Notably, melatonin was shown to reduce the side effects related to of chemo- and radiotherapy and enhance their therapeutic efficacy in advanced cancer patients with poor clinical status, improving the clinical outcome of patients afflicted by cancer. Therefore, this indoleamine potentially presents both curative and palliative actions in the treatment of human neoplasms [97]. Further studies are needed in order to better understand melatonin’s mechanism of action against GBM and to determine which drugs melatonin should or should not be used with as an adjuvant therapy. Furthermore, future studies are needed to evaluate melatonin as a sole antineoplastic therapy, and not just as a supplement to more invasive therapies.

Multidrug resistance is the main defense mechanism developed by GBM against chemotherapy, leading to treatment failure. Aberrant expression and activity of the adenosine triphosphate-binding cassette (ABC) family transporters can induce multidrug resistance in cancer cells [98]. Interestingly, beneficial anti-GBM effects were reported for the combination treatment of melatonin and various drugs, including chemotherapeutic molecules [99]. Melatonin treatment of GBM cell lines and GSCs from surgery specimens increased methylation levels of the ABCG2 promoter, which appears to decrease the transporter expression, causing a synergism between melatonin and chemotherapeutic molecules [100]. The evidence of 5-azacitidine, a DNA methyl-transferase inhibitor, preventing melatonin effects on ABCG2/breast cancer resistance protein (BCRP) expression suggests an epigenetic effect of the indoleamine through the alteration of DNA methyltransferase activity [100].

GBM cells present an elevated expression of Nrf2, contributing to chemoresistance [101,102,103,104,105]. A study by Pan et al. [106] described an increased expression of the Nrf2–antioxidant responsive element (ARE) signaling pathway in TMZ-resistant GBM cells. Treatment with melatonin restored the chemosensitivity of TMZ-resistant GBM cells and increased the rate of cancer cell apoptosis [106]. Pharmacological concentrations of melatonin (1 mM) induced a significant reduction of Nrf2 expression in GBM cells when combined with TMZ [106]. However, a previous study suggested that at low concentration of melatonin may activate the Nrf2–ARE signaling pathway in glioma cells [107]. Further studies are required to confirm the activity of melatonin on the Nrf2–ARE pathway, including in vivo experiments and preclinical studies.

Furthermore, the expression of transcription factor EB (TFEB), a relevant molecule for chemoresistance that transcriptionally regulates several lysosomal genes, is significantly increased in GBM cells [108,109,110]. A recent study from Sung et al. [111] observed whether the combination of vorinostat, a histone deacetylases inhibitor, and melatonin could overcome the effects of TFEB and induce GBM and GSC apoptosis [111].

## 3. In Vivo and Clinical Trial Evidence of Melatonin Effects against Glioblastoma

Despite several in vitro findings of the antineoplastic effect of melatonin against GBM, to date, only a few articles described the effects of melatonin against GBM in animal models, with even fewer describing the effects in humans.

Martín et al. [70] described the efficacy of melatonin treatment in reducing tumor growth in vivo. In particular, glioma cells were pre-treated with melatonin and then injected into rats. Interestingly, at day 11 (six days after the start of the treatment), tumor growth was significantly reduced as compared to the control untreated group. Additionally, at day 14, the reduction in tumor growth reached 50% [70]. It must be mentioned that the concentration used in this study was greater than the concentration used in any human study (15 mg/kg body weight by subcutaneous injection in rats versus 20 mg per day administered orally in clinical trials). Therefore, for future clinical trials, new melatonin dosing schedules equivalent to that used successfully in in vivo studies will hopefully demonstrate the apparent effects of melatonin alone as a antineoplastic agent against GBM.

**Figure 1:**
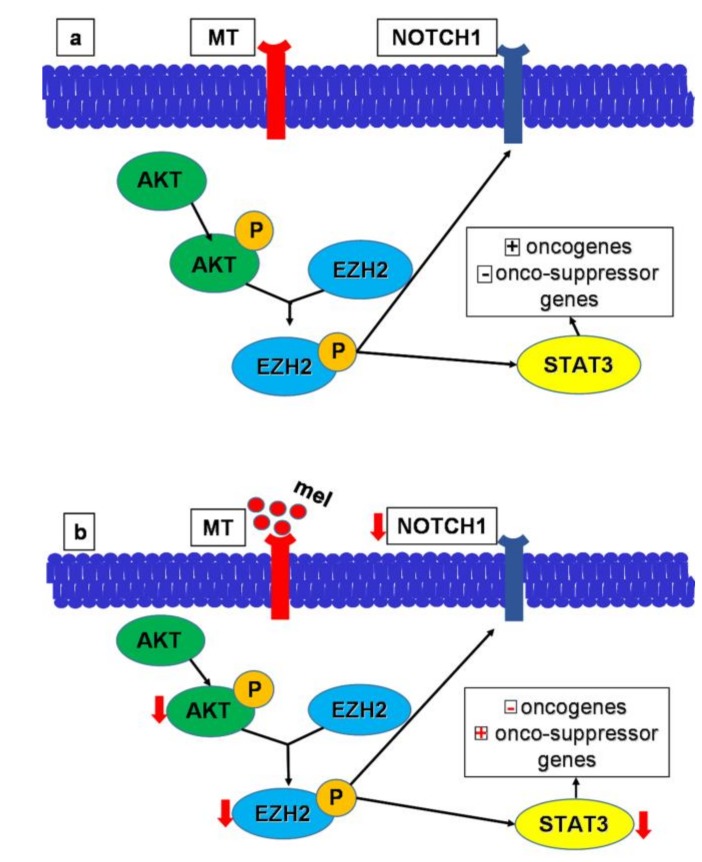
The interaction of melatonin with its membrane receptors induces alterations in the activity of protein kinase B (Akt)–enhancer of zeste 2 polycomb repressive complex 2 subunit (*EZH2*)–signal transducer and activator of transcription 3 (STAT3) and *EZH2*–Notch1 pathways in Akt-overexpressing glioma stem-like cells (GSCs). Melatonin prevents *EZH2* activation and reduces *EZH2* pro-oncotic effects such as the upregulation of Notch1. (**A**) Scheme summarizing the basal activity of Akt–EZH2–STAT3 and EZH2–Notch1 pathways. (**B**) Scheme summarizing the effects induced by melatonin on Akt–EZH2–STAT3 and EZH2–Notch1 pathways. Mel: melatonin; MT: melatonin receptor; P: phosphorylation.

Cancer cell stemness is dynamically influenced by epigenetic mechanisms [112,113,114]. To determine the efficacy of five days of in vitro treatment with melatonin compared with TMZ (5 mmol/L) on GSCs, Chen et al. [16] performed an intracranial injection of GSCs into immunocompromised mice. The control GSCs showed no benefit of TMZ administration, and death usually resulted within 35–45 days. By contrast, the pre-treatment of GSCs with melatonin significantly raised the survival rate. These results suggest that short in vitro melatonin treatment may be able to reduce the number of tumor-initiating GSCs, allowing a delayed tumor development. Notably, Chen et al. [16] determined, for the first time, that the effects of melatonin on GSCs occur mainly through melatonin receptor-dependent signaling. In fact, the expression of GSC stem-cell markers was significantly impaired when GSCs were incubated with luzindole (10 mmol/L), prior to the addition of melatonin. [16]. More specifically, the inhibitory effect of melatonin on GSC growth resulted from alterations in the activity of *EZH2*, rather than its expression levels. *EZH2*, which is activated by protein kinase B (Akt) phosphorylation, catalyzes the methylation of histone H3 and induces signal transducer and activator of transcription 3 (STAT3) [16,115,116,117], which plays a critical role in the cell cycle and other pathways, such as tyrosine phosphorylation, serine phosphorylation, and DNA binding [16,118,119,120,121]. Notably, in GSCs isolated from patients, STAT3 showed specific interactions with *EZH2*, and Chen et al. [16] also observed that melatonin treatment significantly inhibited STAT3 activity. Therefore, by targeting the Akt–*EZH2*–STAT3 axis, melatonin can strongly impair GSC self-renewal and tumor-initiating capacity. Furthermore, Chen et al. [16] also observed that melatonin treatment in Akt-overexpressing GSCs inhibited Akt activation. Additionally, compared to normal human brain specimens, surgical GBM specimens showed a remarkable upregulation of *EZH2* and Notch1 and, as previously reported, Zheng et al. [85] observed that Notch1 was significantly downregulated in knockdown *EZH2* GBM cells, thus underlining the role of melatonin in the inhibition of the *EZH2*–Notch1 signaling axis. Figure 1 summarizes the melatonin mechanism of action on Akt–*EZH2*–STAT3 and *EZH2*–Notch1 signaling pathways.

Notably, the only clinical trial, carried out by Lissoni et al. [58], evaluated the effects of melatonin co-treatment in 30 GBM patients undergoing radical or adjuvant radiotherapy (RT). Patients were randomly divided in groups of RT alone or RT plus 20 mg/day oral melatonin. At one year from the start of the treatment, the patient survival percentage of the RT + melatonin group was significantly higher than the RT alone group (6/14 vs. 1/16 patients). Notably, as previously reported, this clinical trial also described in the RT + melatonin group a decrease in RT and steroid therapy adverse effects, such as infections and alopecia, together with reduced anxiety and improved quality of sleep. Even though future studies with a larger number of patients should be investigated, these results underline the potential utility of melatonin as co-treatment in GBM patients, as well as its capacity to improve patients’ quality of life. Another study from Lissoni et al. [122], tested the combination of melatonin and *Aloe vera*, which has anti-inflammatory effects [123,124,125]. The rationale of the study was to investigate the potential improvement of melatonin antineoplastic effects with the combination of *Aloe vera*. Thus, 50 neoplastic patients with GBM, breast cancer, lung cancer, or gastrointestinal tract tumor, unresponsive to chemotherapy, RT, or endocrine therapy or poor clinical condition precluding chemotherapy, were enlisted. After at least one month following the last chemotherapy session, patients received an oral dose of 20 mg/day of melatonin or the same dose of melatonin plus *Aloe vera* tincture until neoplastic regression. Unfortunately, in the group of melatonin alone, no tumor regression was observed at two months after the start of the treatment, whereas, in the melatonin plus *Aloe vera* group, 8% (two patients out of 24) showed a partial response. However, there were no melatonin-associated adverse events, whereas treatment with *Aloe vera* was associated with diarrhea, even if limited to just the first day of administration [122]. One concern, however, is that in vitro studies typically used a melatonin concentration (1 mM) that may not a be physiologically attainable. As McConnell et al. [94] reported, the concentration of 50 nM closely mimics physiological levels (54 nM) for patients taking 20 mg of melatonin orally, whereas the concentration of 1 mM melatonin may not be physiologically attainable. The use of melatonin as an adjuvant to chemotherapy showed promising results, both improving the efficacy of treatment and reducing side effects [17,94]. Clinical trials studying melatonin-integrated therapies in oncological patients, with tumors other than GBM, are generally conducted outside clinical guidelines after failing to respond to the gold-standard therapy and with an already compromised prognosis. This is certainly a limit in the study of a potential efficacy of melatonin treatment in increasing the healing rate or the tumor regression in such patients. Even with the evidence of antineoplastic effects in vitro and palliative effects in humans, the integration of this multitasking indoleamine in the first-line therapy of a malignancy would face logical ethical issues. However, improving quality of sleep and reducing the side effects from oncological therapies is already a very important result in critically ill patients that should not be undervalued [58,59,69].

Clinical treatment with melatonin faces some drawbacks, such as irregular absorption and a short circulating half-life [126,127]. In order to avoid these obstacles, melatonergic agonists were developed and used for sleep dysregulation and depression [128,129]. In comparison to melatonin, ramelteon and agomelatine, both melatonin receptor agonists, have significant advantages through higher receptor affinity, higher absorption concentration, and longer half-life. Agomelatine, licensed by the European Medicines Agency, is a melatonin agonist used in the treatment of depression [130]. It shows a higher affinity to melatonergic receptors, with longer half-life, and it better penetrates the blood–brain barrier [129,131,132]. Ramelteon, approved by the Food and Drug Administration of the United States (FDA), is an MT1 and MT2 agonist free of side effects and available for altered sleep initiation [133,134,135]. The affinity of this molecule to MT1 and MT2 is remarkably higher with respect to melatonin and shows improved oral absorption and circulating half-life [136]. These emerging findings suggest that melatonergic agonists present potentially essential roles as an approach against GBM. Recently, Zhou et al. [137] reported that agomelatine, also in addition to melatonin, may act as a common upstream signal between autophagy and apoptosis of GBM cells, with the effect attenuated by treatment with luzindole. These results may lead to the development of new therapeutic strategies for glioma.

Furthermore, using the ionic gelation method, chitosan/tripolyphosphate nanoparticles (CSNPs) were prepared with a size range from 115 to 740 nm in order to overcome the pharmacokinetic limitations of melatonin and improve its efficacy against GBM. CSNPs were tested in the human GBM cell line U87MG. The use of small-size particles as a carrier system led to higher cellular uptake and blood vessel penetration. GBM cells showed higher melatonin CSNP uptake compared to non-malignant cells [138]. The uptake of these nanoparticles depends on the cell surface charge and the metabolic activity of cancerous cells [139]. Previous studies showed that blank CSNPs increased ROS concentration in cancerous cells, leading to mitochondrial cytochrome c release and apoptosis through the caspase cascade [140]. Recently, de Oliveira Junior et al. [141] described, for the first time, the application of nanoparticle-mediated nose-to-brain delivery of melatonin for the treatment of GBM both in vitro and in vivo. Pharmacokinetic data revealed an increased melatonin concentration in the brain of rats administered intranasally with melatonin nanoparticles compared to melatonin suspension, in both nasally and orally administered groups. Furthermore, the nanoencapsulation of melatonin was crucial to promoting selective and strong cytotoxic effects on the U87MG human GBM cell line.

GBM cells were incubated with equivalent doses of melatonin and melatonin-loaded chitosan. Loading of blank nanoparticles without melatonin was evaluated as control. Both melatonin and melatonin-loaded chitosan reduced GBM cell viability with a marked time- and dose-dependent pattern [138]. Interestingly, the study described higher anticancer activity in the melatonin CSNP group than in the “pure” melatonin cells, leaving only 22% against 42% of viable cells after 24 h of incubation. With longer exposure (48 h and 72 h), the “pure” melatonin group did not show further reduction in the percentage of cell viability. This evidence suggests that melatonin CSNPs extend the melatonin anticancer activity with respect to “pure” melatonin. Differences in cellular metabolism and surface charge may explain the distinctive response to melatonin CSNPs between GBM cells and control cells [138].

## 4. Conclusions

**Figure 2:**
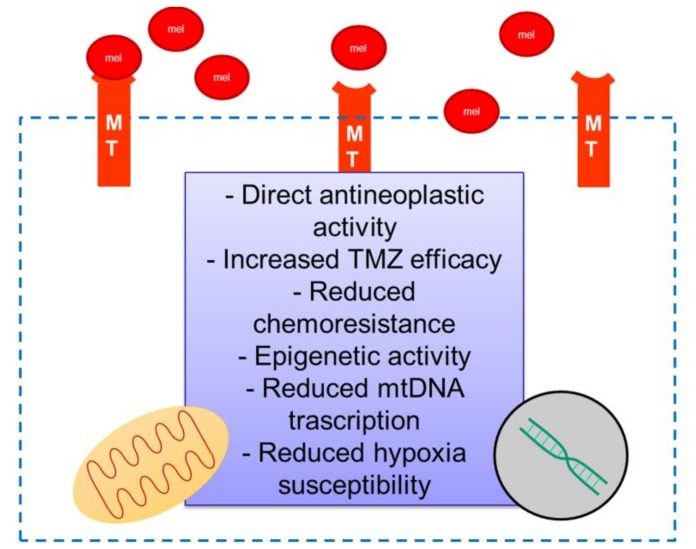
Melatonin may induce, in glioblastoma (GBM) cells, an intracellular transcription cascade, which, in turn, leads to an antineoplastic response reducing/blocking oncogene expression, chemoresistance, and other cellular mechanisms. Mel: melatonin; MT: melatonin receptor; mtDNA: mitochondrial DNA; TMZ: temozolomide.

Melatonin is a promising agent in the field of antineoplastic research, and this multitasking indoleamine shows an incredible variety of properties against GBM (Figure 2). As described in this review, melatonin specifically interacts with cancer cells and interferes with GBM proliferative activity and aggressiveness. In vitro studies investigating the effects of GBM cells through therapy of melatonin in association with the standard chemotherapy TMZ showed relevant and notable antineoplastic effects together with contrasting TMZ chemoresistance. The association of melatonin with chemotherapy may enhance the cytotoxic effects against cancer and help to decrease its dosage, thereby reducing side effects and improving quality of life. Further studies observed also interesting epigenetic activity of melatonin against GBM. In addition, local drug delivery techniques or the improvement of the pharmacokinetics through melatonin-like molecules could achieve effective clinical outcomes. Despite the remarkable in vitro evidence of melatonin efficacy against GBM, only a few in vivo articles and even fewer clinical trials analyzed this specific topic. A scientific evidence consortium with in vivo and especially preclinical studies could be the first step toward the potential application of melatonin against GBM, as, at least, a combined therapy with TMZ.
